# A Sequence-Dependent Combination of Photodynamic Therapy and Carboxyamidotriazole Orotate for Enhanced Treatment of Glioblastoma

**DOI:** 10.3390/ijms27136091

**Published:** 2026-07-07

**Authors:** Jiaxing Qiu, Yunfan Li, Jiaming Zou, Yucheng Wang, Rui Ju, Lei Guo

**Affiliations:** Department of Pharmacology, Institute of Basic Medical Sciences & School of Basic Medicine, Chinese Academy of Medical Sciences & Peking Union Medical College, Beijing 100005, China; qiujx@student.pumc.edu.cn (J.Q.); liyunfan0909@163.com (Y.L.); zoujiaming5@163.com (J.Z.); jurui@ibms.pumc.edu.cn (R.J.)

**Keywords:** glioblastoma, photodynamic therapy, carboxyamidotriazole orotate, sequence-dependent interaction, metabolic inhibition

## Abstract

Glioblastoma (GBM) remains a highly lethal malignancy characterized by profound treatment resistance and metabolic plasticity. While photodynamic therapy (PDT) and the mitochondrial complex I inhibitor carboxyamidotriazole orotate (CTO) have individually shown promise, their combined potential requires further exploration and optimization. This study systematically investigated the interaction between 5-aminolevulinic acid (5-ALA)-mediated PDT and CTO in U87 GBM models. Intriguingly, we discovered a sequence-dependent interaction under the tested treatment schedules: CTO pre-incubation before PDT resulted in attenuated PDT-induced cytotoxicity, possibly due to CTO-mediated suppression of reactive oxygen species (ROS) accumulation. In contrast, a sequential “PDT→CTO” regimen enhanced anti-tumor efficacy both in vitro and in vivo. Mechanistically, the sequential approach was associated with enhanced mitochondrial depolarization and reduced expression of glycolysis-related genes, suggesting a potential metabolic “dual-hit” involving disturbance of mitochondrial function and compensatory glycolytic adaptation. These results highlight treatment sequence as a critical determinant of PDT-CTO interaction and provide a basis for further preclinical investigation of PDT followed by metabolic intervention as a combination strategy with potential translational relevance.

## 1. Introduction

Glioblastoma, IDH-wildtype (GBM), as defined in the 2021 WHO Classification of Tumors of the Central Nervous System, remains one of the most lethal and aggressive primary brain malignancies in adults, characterized by a highly infiltrative growth pattern and a dismal prognosis [1,2,3]. The current clinical standard of care, known as the Stupp protocol, consists of maximal safe surgical resection followed by synchronous radiotherapy and chemotherapy with temozolomide (TMZ) [4,5]. Despite these intensive interventions, the prognosis of GBM remains poor with a median survival of 15~18 months. Therapeutic failure is partly attributed to limited drug penetration across the blood–brain barrier (BBB), and an immunosuppressive tumor microenvironment (TME) that promotes immune evasion and treatment resistance [6,7,8].

Photodynamic therapy (PDT) offers a compelling solution to these dilemmas [9,10,11]. As a minimally invasive and repeatable intervention, PDT uses a relatively non-toxic photosensitizer (PS) and localized light of a specific wavelength to generate reactive oxygen species (ROS), inducing oxidative damage to tumor cells, with the ROS profile and therapeutic efficacy influenced by oxygen availability [12,13]. Direct optical-fiber-mediated irradiation allows PDT to exert localized cytotoxic effects at the tumor site, thereby reducing the need for systemic tumor targeting and partially bypassing the delivery barriers associated with the BBB. Moreover, due to a unique metabolic trait, GBM cells selectively accumulate protoporphyrin IX (PpIX) intracellularly after 5-aminolevulinic acid (5-ALA) administration [14,15]. Since 5-ALA is already clinically approved for fluorescence-guided surgery (FGS) to visualize tumor margins, PDT serves as a natural and seamless therapeutic extension to eradicate residual infiltrative cells in the surgical cavity [14]. Crucially, PDT can induce immunogenic cell death (ICD), triggering the release of tumor-associated antigens (TAAs) and damage-associated molecular patterns (DAMPs) [16]. This process promotes dendritic cell maturation and cytotoxic T lymphocytes (CTLs) activation, potentially transforming the “cold” GBM TME into a “hot” immune-responsive environment [17].

Despite these advantages, the clinical efficacy of PDT in GBM remains hindered by several critical bottlenecks. The red light commonly used in clinical practice (typically around 630 nm) has limited penetration depth in tissues [18]. The significantly hypoxic TME of GBM also drastically reduces the efficiency of the oxygen-reliant Type II photodynamic reaction [19]. Moreover, GBM’s high metabolic plasticity enables tumor cells to adapt to stress by shifting between metabolic pathways, increasing tolerance to interventions [20,21]. To overcome these hurdles, developing combination therapeutic strategies has become a vital direction.

Carboxyamidotriazole orotate (CTO)—the orotate salt form of carboxyamidotriazole (CAI)—has emerged as a promising candidate for GBM treatment. Extensive preclinical studies have demonstrated that CAI can regulate tumor signaling networks and inhibit growth and metastasis across various cancer models [22,23,24]. Notably, CTO achieved remarkable results in the clinical trial of GBM. The combination of CTO and TMZ in both newly diagnosed and recurrent GBM patients revealed favorable safety profiles and encouraging anti-tumor efficacy [25,26]. As a non-cytotoxic small molecule compound, CTO has multiple functions in metabolism and immune regulation, enabling it to play a synergistic role in various combined treatment regimens. Mechanistically, CTO acts as a specific inhibitor of mitochondrial respiratory chain complex I, thereby reducing oxidative phosphorylation (OXPHOS) [27,28]. By suppressing the oxygen consumption rate, CTO increases the overall oxygen availability within the TME, thereby alleviating hypoxia. Beyond metabolic effects, CTO plays a crucial role in immune remodeling, such as modulating the polarization of tumor-associated macrophages (TAMs) [29]. Based on these findings, we hypothesized that the combination of CTO and PDT could yield synergistic benefits. Mechanistically, CTO-mediated modulation of tumor hypoxia and metabolism may improve the responsiveness of GBM to PDT, whereas PDT may complement CTO through spatially confined cytotoxic effects.

In this study, we investigated the therapeutic feasibility of combining CTO with 5-ALA-mediated PDT for GBM. The human U87 cell line, a widely used experimental model in GBM research, served as the primary system for both in vitro and in vivo evaluation. Our findings revealed a sequence-dependent interaction between these two treatments: while CTO pre-incubation before PDT led to antagonistic effects, administration of CTO after PDT enhanced antitumor efficacy in vitro and significantly suppressed tumor growth in vivo. This enhanced effect was accompanied by greater mitochondrial depolarization and reduced expression of glycolysis-related genes, suggesting that the sequential “PDT→CTO” regimen may exploit tumor metabolic vulnerabilities by intensifying mitochondrial dysfunction while limiting glycolysis-associated compensatory responses. This study provides a rationale for potential sequence-dependent combination therapy of GBM.

## 2. Results

### 2.1. Optimization of PDT Parameters in U87 Cells

To establish the in vitro PDT conditions for subsequent experiments, we first optimized the concentration of 5-ALA, a precursor of PpIX, and the light dose. U87 cells were incubated with increasing concentrations of 5-ALA (0~1000 µM) and then exposed to 635 ± 5 nm light at a fixed irradiance of 30 mW/cm^2^ for 0, 150, or 300 s, corresponding to fluences of 0, 4.5, and 9.0 J/cm^2^, respectively. In the absence of light, 5-ALA showed no significant dark toxicity at concentrations up to 250 µM (Figure S1A). However, irradiation induced a marked decrease in cell viability in a 5-ALA concentration- and light dose-dependent manner (Figure S1A). Specifically, treatment with 100 µM 5-ALA plus 4.5 J/cm^2^ irradiation or 25 µM 5-ALA plus 9.0 J/cm^2^ irradiation reduced cell viability to below 50% of the untreated control. Considering the shorter irradiation time and comparable phototoxic efficacy, 100 µM 5-ALA followed by 4.5 J/cm^2^ laser irradiation was selected as the working PDT condition for subsequent experiments unless otherwise specified. The working concentration of 100 μM was considered safe with respect to dark toxicity (Figure S1A).

Furthermore, the incubation time required for optimal intracellular PpIX accumulation under incubation with 100 μM 5-ALA was investigated using confocal microscopy. The metabolic kinetics of PpIX exhibited a dependence on oxygen levels. Confocal imaging and quantitative analysis of PpIX fluorescence intensity showed that robust PpIX accumulation was detectable after 2 h of incubation under normoxia (21% O_2_), whereas PpIX accumulation was delayed under 5% O_2_, requiring 4~6 h to reach comparable levels (Figure S1B,C). Based on biological and technical considerations, 5% O_2_ was selected as a standardized moderately hypoxic condition for subsequent in vitro experiments. Severe hypoxia may reduce 5-ALA-induced PpIX biosynthesis and weaken oxygen-dependent PDT reactions [30,31]. In contrast, 5% O_2_ provided a reproducible hypoxia setting in which PpIX generation and PDT cytotoxicity remained measurable, while still imposing tumor-relevant oxygen limitation [32,33]. In addition, a 4 h 5-ALA incubation was performed to ensure sufficient PpIX generation.

### 2.2. CTO Pre-Incubation Followed by PDT Results in Antagonistic Anti-Tumor Effects

**Figure 1:**
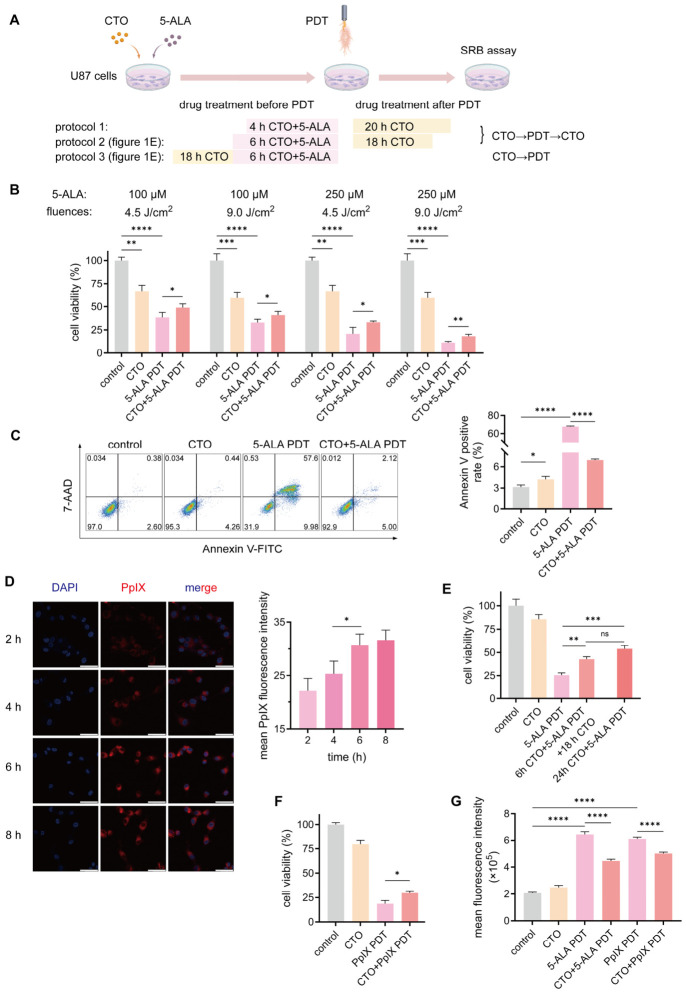
CTO pre-incubation before PDT exhibits antagonistic anti-tumor effects. (**A**) Schematic diagram of the treatment regimens in U87 cells (created with BioGDP.com). Protocols 1 and 2 represent “CTO→PDT→CTO” regimens, with CTO/5-ALA co-incubation for 4 or 6 h before PDT followed by CTO exposure after PDT for 20 or 18 h, respectively; protocol 3 represents a “CTO→PDT” regimen, where the entire 24 h CTO exposure occurred before PDT, comprising 18 h of CTO treatment followed by 6 h of CTO/5-ALA co-incubation. (**B**) Cell viability of U87 cells treated according to protocol 1 using 5-ALA-mediated PDT at the indicated 5-ALA concentrations and light fluences. (**C**) Representative flow cytometric plots and quantitative analysis of cell death in U87 cells using Annexin V-FITC/7-AAD dual staining. Colors in the pseudocolor plots indicate event density, ranging from blue (low density) to red (high density). (**D**) Representative confocal fluorescence images and quantitative analysis of intracellular PpIX accumulation in U87 cells co-treated with 5-ALA and CTO under hypoxia (5% O_2_) across indicated time intervals (2~8 h). DAPI-stained nuclei are shown in blue, PpIX fluorescence is shown in red, and the merged images represent an overlay of the two channels. PpIX fluorescence intensity was quantified from confocal images using ImageJ (version 1.51) and expressed as mean fluorescence intensity (MFI). Scale bar: 50 μm. (**E**) Cell viability of U87 cells under protocol 2 and 3. (**F**) Cell viability of U87 cells treated with CTO and exogenous PpIX-mediated PDT. Cell viability in (**B**,**E**,**F**) was measured by SRB assay and normalized to the untreated control group. (**G**) Total intracellular ROS levels measured by DCFH-DA staining and flow cytometry, expressed as MFI. Data are presented as mean ± SD (*n* = 3). * *p* < 0.05, ** *p* < 0.01, *** *p* < 0.001, **** *p* < 0.0001, ns: non-significant (one-way ANOVA followed by Tukey’s post hoc test).

To evaluate the efficacy of combining CTO with PDT, we first evaluated a “CTO→PDT→CTO” regimen, in which CTO was administered during the 4 h 5-ALA incubation before irradiation and then re-administered for 20 h after PDT (Figure 1A). Unexpectedly, SRB assays revealed that the cell viability in the combined treatment group was significantly higher than that in the PDT monotherapy group (Figure 1B). Apart from the working PDT condition we chose, this antagonistic effect was consistently observed across different 5-ALA concentrations (100 µM and 250 µM) and different light exposures (4.5 J/cm^2^ and 9.0 J/cm^2^) (Figure 1B). Furthermore, the antagonistic effect was also consistently validated in two additional GBM cell lines, U251 and GL261, under the working PDT condition (Figure S2).

To further characterize this antagonism, we assessed cell death by Annexin V-FITC/7-AAD double staining. While PDT alone induced Annexin V positivity in approximately 68% of U87 cells, the addition of CTO dramatically reduced this proportion (Figure 1C). These results suggest that CTO interferes with the PDT-initiated cell death response, potentially involving multiple pathways, such as apoptosis, necroptosis, and ferroptosis [34,35].

We initially hypothesized that CTO might hinder the metabolic conversion of 5-ALA to the active photosensitizer PpIX. Confocal microscopy, together with quantitative analysis of intracellular PpIX fluorescence intensity, showed that, relative to the CTO-free condition (Figure S1B,C), CTO delayed intracellular PpIX accumulation, requiring 6 h to reach a stable level. Nevertheless, the final fluorescence intensity was not altered (Figure 1D). Extending the pre-PDT CTO/5-ALA co-incubation time to 6 h to allow more PpIX accumulation failed to restore PDT-mediated cytotoxicity (Figure 1A,E). We also introduced a “CTO→PDT” regimen in which the 5-ALA incubation time was maintained at 6 h, while the entire 24 h CTO exposure was shifted to the pre-PDT period. The antagonistic effect remained evident under this condition (Figure 1E). Furthermore, when exogenous PpIX was used directly as the PS to bypass 5-ALA metabolism, CTO continued to attenuate PDT-mediated cytotoxicity (Figure 1F).

Since PDT-mediated cell death relies heavily on the production of ROS, we measured total ROS levels using the DCFH-DA probe. Pre-treatment with CTO significantly reduced the DCFH-DA fluorescence intensity induced by either 5-ALA or PpIX-mediated PDT (Figure 1G), demonstrating a suppressed ROS accumulation. However, due to the limited specificity of DCFH-DA, these results indicate reduced overall oxidative stress rather than a specific ROS source.

### 2.3. Sequential “PDT→CTO” Treatment Synergistically Enhances In Vitro Anti-Tumor Efficacy

Given the antagonistic effects observed in the treatment regimens of “CTO→PDT →CTO” and “CTO→PDT”, we hypothesized that avoiding CTO pre-incubation and shifting CTO administration to the post-PDT period might circumvent this antagonism and improve the therapeutic outcome. We therefore designed a sequential “PDT→CTO” regimen, where U87 cells were first subjected to the established PDT condition (100 µM 5-ALA for 4 h followed by 4.5 J/cm^2^ irradiation) and subsequently treated with CTO for 24 h.

**Figure 2:**
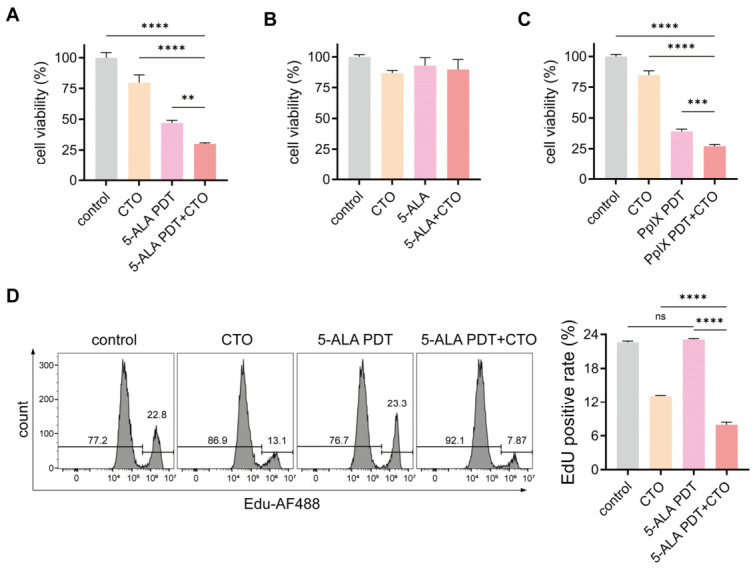
Sequential “PDT→CTO” treatment inhibits proliferation in U87 cells. (**A**) Cell viability of U87 cells treated with the sequential “5-ALA PDT→CTO” regimen. (**B**) Cell viability of U87 cells treated with 5-ALA and CTO without light irradiation. (**C**) Cell viability of U87 cells treated with the sequential “PpIX PDT→CTO” regimen. Cell viability in (**A**–**C**) was measured by SRB assay and normalized to the untreated control group. (**D**) Representative flow cytometric histograms and statistical analysis of cell proliferation measured by EdU incorporation. Data are expressed as mean ± SD (*n* = 3). ** *p* < 0.01, *** *p* < 0.001, **** *p* < 0.0001, ns: non-significant (one-way ANOVA followed by Tukey’s post hoc test).

SRB assays demonstrated that this sequential regimen significantly enhanced cytotoxicity compared to PDT monotherapy, resulting in an additional 15% reduction in cell viability (Figure 2A). The Bliss single-point synergy score at the tested doses of PDT and CTO was 0.076 (> 0), indicating a possible synergistic trend. To determine whether this effect was specifically mediated by the photodynamic reaction, we tested a “5-ALA + CTO” combination without light exposure. This group showed substantially lower cytotoxicity than the sequential “PDT→CTO” regimen, suggesting that the synergy is dependent on photodynamic reaction rather than the mere presence of 5-ALA (Figure 2B). Consistent synergistic results were also observed when PpIX was utilized as the PS (Figure 2C). At the tested dose, the Bliss single-point synergy score was 0.062 (>0), also suggesting a synergy. Furthermore, the synergistic effect of the sequential “PDT→CTO” regimen was recapitulated in U251 and GL261 cells (Figure S3).

The anti-proliferative capacity of the sequential treatment was further evaluated using the EdU incorporation assay. While CTO monotherapy exhibited a clear inhibitory effect on U87 cell proliferation, the “PDT→CTO” group produced a significantly greater suppression of EdU incorporation than CTO alone (Figure 2D). Notably, PDT monotherapy caused a slight, non-significant increase in cell proliferation, which is consistent with its role in inducing acute cell death rather than long-term growth inhibition [34,35].

### 2.4. Sequential “PDT→CTO” Treatment Exhibits Potent Anti-Tumor Efficacy In Vivo

**Figure 3:**
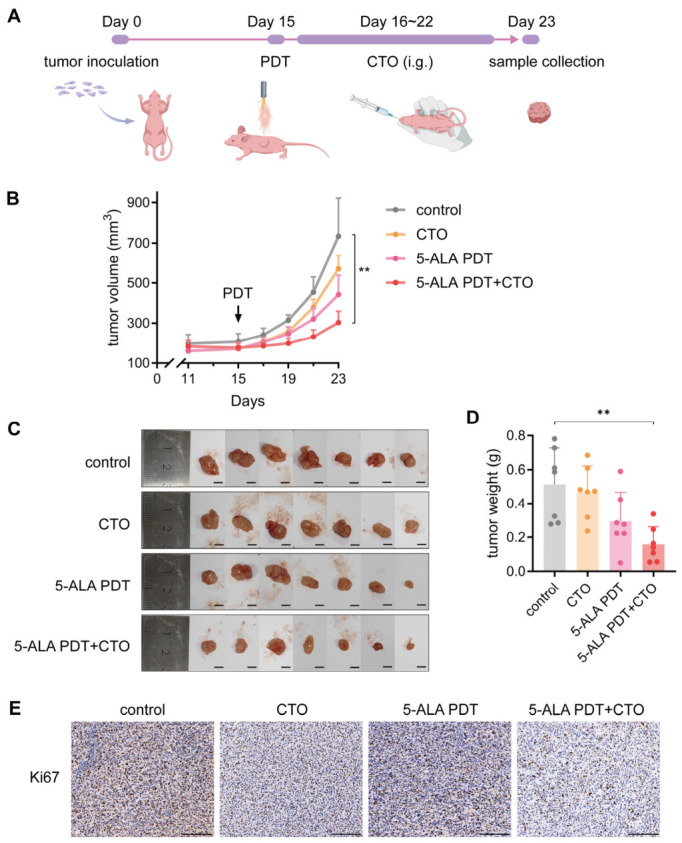
Synergistic antitumor efficacy of sequential “PDT→CTO” treatment in the U87 subcutaneous xenograft model. (**A**) Schematic representation of the experimental design and treatment protocol for the U87 subcutaneous xenograft model in BALB/c nude mice (created with BioGDP.com). Tumor cells were inoculated on day 0. Mice received a single-dose PDT treatment on day 15, followed by daily CTO intragastric administration from days 16 to 22. Tumors were collected on day 23. (**B**) Monitoring of tumor volume during the experiment. Tumor volume was calculated as V = (length × width^2^)/2. (**C**) Representative photographic images of harvested tumors at the end of the study. Scale bar: 5 mm. (**D**) Comparison of mean tumor weights at the end of the study. (**E**) Representative IHC staining of Ki67 expression in U87 subcutaneous tumor sections. Scale bar: 50 μm. Data are expressed as mean ± SD (*n* = 7). ** *p <* 0.01 (one-way ANOVA followed by Tukey’s post hoc test).

To validate the therapeutic potential of the sequential regimen in vivo, we established a GBM xenograft model in BALB/c nude mice using U87 cells. Following the optimized “PDT→CTO” protocol, mice in the combination group received a single-dose PDT treatment on day 15, followed by daily CTO administration (90 mg/kg, i.g.) (Figure 3A). The treatment was well-tolerated; mean body weights across all groups showed no significant differences by the end of the study, indicating a favorable safety profile (Figure S4). During the in vivo experiment, gross examination did not reveal obvious local tissue damage in areas shielded from laser irradiation, suggesting no overt macroscopic dark toxicity under the present treatment conditions.

The sequential combination therapy demonstrated superior anti-tumor activity compared to either monotherapy. While CTO and PDT monotherapies had certain tumor suppression effects, the sequential “PDT→CTO” treatment significantly suppressed tumor progression with a tumor growth inhibition (TGI) rate of 58.7% (Figure 3B,C). The Bliss single-point synergy score calculated from the endpoint TGI values was 0.063 (>0), suggesting a synergistic trend. This potent efficacy was further corroborated by tumor weight measurements (Figure 3D). Immunohistochemical (IHC) staining for Ki67, a marker of cells in the cell cycle, revealed a noticeable reduction in cycling tumor cells within the combination group, suggesting a potential impairment of proliferative capacity (Figure 3E). These results suggest that the sequential “PDT→CTO” regimen synergistically inhibits tumor growth in vivo.

### 2.5. Mechanistic Insights into Sequential “PDT→CTO” Treatment

**Figure 4:**
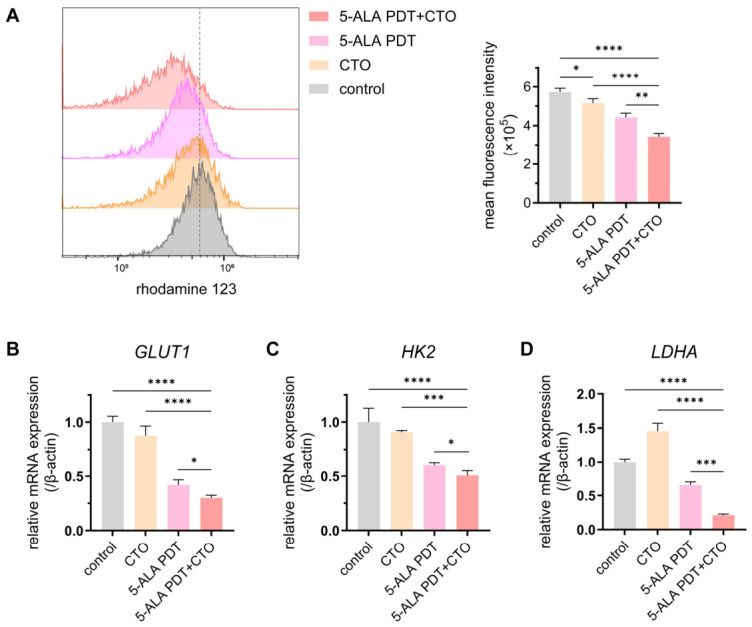
Sequential “PDT→CTO” treatment induces mitochondrial depolarization and reduces glycolysis-related gene expression in U87 cells. (**A**) Representative flow cytometric histograms and statistical analysis of mitochondrial membrane potential measured by rhodamine 123 staining. The dashed vertical line marks the reference fluorescence position of the control peak. (**B**–**D**) Relative mRNA expression levels of key glycolysis-related genes, including *GLUT1* (**B**), *HK2* (**C**), and *LDHA* (**D**), in U87 cells after “PDT→CTO” treatment, as determined by RT-qPCR. mRNA expression was normalized to β-actin and calculated using the 2^−ΔΔCt^ method, with the expression level in the control group defined as 1. Data are expressed as mean ± SD (*n* = 3). * *p* < 0.05, ** *p* < 0.01, *** *p* < 0.001, **** *p* < 0.0001 (one-way ANOVA followed by Tukey’s post hoc test).

Given that both PDT and CTO have been associated with mitochondrial perturbation, we first examined mitochondrial membrane potential (MMP), an important indicator of mitochondrial functional status, to further elucidate the mechanisms underlying the synergistic anti-tumor effects of the sequential “PDT→CTO” regimen. MMP was assessed by rhodamine 123 staining and flow cytometry. Either PDT or CTO monotherapy reduced fluorescence intensity, whereas sequential “PDT→CTO” treatment caused a more pronounced leftward shift in the fluorescence histogram and a significantly lower mean fluorescence intensity than either monotherapy (Figure 4A). These results indicate that the “PDT→CTO” regimen induces mitochondrial depolarization, consistent with impaired mitochondrial function.

Because tumor cells may rely on glycolytic compensation when mitochondrial function is impaired, we next examined whether the sequential “PDT→CTO” regimen affected glycolysis-related gene expression. Compared with monotherapy, the “PDT→CTO” regimen significantly downregulated the mRNA levels of several glycolysis-associated markers, including the glucose transporter 1 (*GLUT1)*, hexokinase 2 (*HK2*), and lactate dehydrogenase A (*LDHA*) (Figure 4B–D). These findings indicate that the combined therapy may affect glycolytic adaptation.

## 3. Discussion

In this study, we explored the therapeutic potential of combining CTO with 5-ALA-mediated PDT for GBM, a highly treatment-resistant malignancy. CTO exposure before irradiation antagonized PDT-mediated cytotoxicity. This effect was accompanied by reduced PDT-induced ROS accumulation, suggesting that decreased oxidative stress may be involved in the attenuation of PDT efficacy by CTO. Conversely, the sequential “PDT→CTO” treatment demonstrated synergy, significantly reducing cell viability and proliferation in vitro and suppressing tumor growth in vivo. Our findings provide the first evidence that the efficacy of this combination is strongly sequence-dependent, highlighting a potential strategy for enhancing the anti-tumor effects of both modalities against GBM.

Our data showed that CTO antagonized PDT efficacy whenever it was present before irradiation. This finding was unexpected, because inhibition of mitochondrial respiration would generally be presumed to increase local oxygen availability and thereby favor oxygen-dependent PDT. One possible explanation is that CTO-induced mitochondrial complex I inhibition may alter intracellular redox-associated responses and counteract PDT-induced oxidative stress. In our previous work, CTO was shown to promote NADH accumulation by limiting its oxidation to NAD^+^ [28]. Elevated NADH may interfere with PpIX-mediated photochemistry through several mechanisms. On the one hand, NADH may donate electrons to ^3^PpIX* and shift the photodynamic reaction toward a Type I pathway, thereby altering the ROS profile, with a possible reduction in highly cytotoxic ^1^O_2_ generation [36,37]. On the other hand, NADH may act as an endogenous quencher of excited PpIX, possibly shortening its excited-state lifetime and limiting its reaction with oxygen [38,39]. For instance, a kinetic study showed that NADH reductively quenched excited zinc protoporphyrin IX with a rate constant of 6.3 × 10^5^ L/(mol·s) [40]. In addition, although NADPH is the primary cofactor for the glutathione and thioredoxin systems, NADH also contributes to maintaining the intracellular reducing environments [41]. Thus, CTO-induced NADH accumulation may weaken PDT efficacy by reducing ROS generation and enhancing reductive buffering capacity. In line with this view, depletion of intracellular reducing agents (such as GSH, NADPH and NADH), has been reported to sensitize tumors to PDT treatment [37,42,43,44]. Further studies directly measuring mitochondrial ROS, ^1^O_2_ generation, and antioxidant buffering capacity are needed to clarify whether CTO influences PDT-associated redox responses.

More broadly, these findings add a layer of complexity to the conventional view that relieving hypoxia in the TME should necessarily enhance PDT efficacy [19]. In our models, the reduced PDT response following mitochondrial inhibition suggests that any potential benefit from increased oxygen availability may be counterbalanced by other cellular effects, possibly involving changes in redox regulation. Notably, recent studies have moved beyond the strategy of inhibiting mitochondrial respiration alone by combining mitochondrial respiration inhibitors with agents that deplete cellular reductive capacity (such as glutathione-depleting agents), thereby promoting ROS accumulation during PDT [45,46,47]. For example, co-delivery of S-nitrosated human serum albumin (HSA-SNO) with a PS not only consumes GSH but also releases nitric oxide to inhibit mitochondrial respiration, thereby synergistically enhancing the PDT efficacy [46].

In this context, rather than introducing additional agents to counteract reductive capacity, our research points to an alternative solution through temporal optimization. The sequential “PDT→CTO” regimen could bypass the antagonistic window by allowing PDT-induced oxidative damage to occur before CTO administration. Under this regimen, PDT may serve as the “instantaneous blow,” where photochemically generated ROS could rapidly damage mitochondrial structures and functions. Subsequently, continuous CTO exposure may further enhance mitochondrial stress through inhibition of mitochondrial complex I. Consistent with this interpretation, the sequential “PDT→CTO” treatment induced a more pronounced reduction in MMP than either monotherapy, suggesting greater mitochondrial dysfunction. Tumor cells typically respond to impaired mitochondrial respiration by activating compensatory mechanisms to maintain ATP production and support survival [48,49]. In our experimental setting, the sequential “PDT→CTO” was accompanied by significantly reduced mRNA expression of several key glycolytic genes, including *GLUT1*, *HK2*, and *LDHA*, compared to the monotherapy groups. Functionally, GLUT1 mediates glucose uptake across the plasma membrane, HK2 catalyzes the first step of glycolysis by phosphorylating glucose [50], and LDHA promotes the conversion of pyruvate to lactate while regenerating NAD^+^to sustain glycolytic flux [51]. These changes indicate that the sequential regimen may suppress glycolysis responses at multiple points. Together, “PDT→CTO” treatment may impose mitochondrial stress and limit glycolysis-related compensation, potentially causing a metabolic “dual-hit” to GBM cells. Nevertheless, this interpretation remains a working model, and direct measurements of mitochondrial respiratory activity, glycolytic flux, ATP production, glucose uptake, and lactate release are needed to further validate this proposed metabolic mechanism.

In the in vivo study, we adopted a single PDT session followed by seven consecutive days of CTO administration, rather than repeated “PDT→CTO” cycles. This schedule reflects the distinct translational features of the two modalities: 5-ALA-mediated PDT is plausibly positioned as a localized intraoperative adjunct to 5-ALA FGS, with the goal of eradicating residual infiltrative tumor cells in the resection cavity [10,52], whereas CTO is an orally available agent that can be administered repeatedly to sustain drug exposure [23,25,53]. Repeated PDT would require additional photosensitizer administration and optical irradiation procedures, potentially increasing procedural complexity. Therefore, the present regimen was designed as a clinically practical treatment module. Whether repeated “PDT→CTO” modules could further enhance antitumor efficacy while maintaining acceptable safety and procedural feasibility warrants investigation in future studies.

Several limitations of this study should be addressed. First, this study used established cell-line-based models, with U87 serving as the principal model. U87 is well-established and remains widely used for reproducible proof-of-concept studies. However, as a long-term cultured cell line, it does not fully represent the heterogeneity of patient-derived, molecularly characterized GBM. In particular, the commercially distributed U87 line has been reported to genetically differ from the original Uppsala line [54], and some xenograft histological features may differ from those of human glioblastoma [55,56]. Moreover, the subcutaneous U87 xenograft model in nude mice does not recapitulate several key features of the GBM microenvironment, including the BBB, brain extracellular matrix, and cellular interactions with microglia and other brain-resident cells. Therefore, further validation in molecularly characterized patient-derived cultures, orthotopic models, organoids, or patient-derived xenografts would help establish the translational relevance of the “PDT→CTO” strategy. Second, our in vitro experiments were performed under a single moderate hypoxia condition of 5% O_2_. However, patient GBMs exhibit heterogeneous oxygen gradients, ranging from relatively oxygenated perivascular regions to severely hypoxic or near-anoxic necrotic areas [57,58,59]. Therefore, responses observed under 5% O_2_ may not fully represent the PDT-CTO interaction across all oxygen niches, particularly under extremely hypoxic conditions where oxygen availability may become the dominant limiting factor for PpIX production or PDT-mediated ROS generation [30,31]. Third, we used single-point Bliss synergy scores as an exploratory measure of PDT and CTO interaction. Although positive Bliss scores support a synergistic tendency of the “PDT→CTO” regimen under the tested conditions, they cannot substitute for comprehensive synergy analyses based on full dose–response matrices. Future studies using Chou–Talalay analysis, response surface modeling, or isobolographic approaches are needed to define the synergy landscape and optimize dosing strategies.

## 4. Materials and Methods

### 4.1. Reagents

Carboxyamidotriazole orotate (CTO), 5-aminolevulinic acid (5-ALA), and protoporphyrin IX (PpIX) were purchased from MedChemExpress (Shanghai, China). Dimethyl sulfoxide (DMSO) was purchased from VWR Chemicals, LLC (Solon, OH, USA). CTO was dissolved in DMSO to a 20 mM stock and diluted to a final concentration of 10 μM for treatment. 5-ALA and PpIX were also dissolved in DMSO to 500 mM and 2 mM stock solutions, respectively, and diluted to specified concentrations for cell treatment.

### 4.2. Cell Culture

Human U87 MG (U87) and U251 MG (U251) glioblastoma cell lines, as well as murine GL261 glioblastoma cell line, were obtained from the Cell Resource Center of the Institute of Basic Medical Sciences, Chinese Academy of Medical Sciences, Beijing, China. U87, U251 and GL261 cells are all characterized as IDH-wildtype. High-glucose DMEM and phosphate-buffered saline (PBS) were obtained from the Cell Resource Center of the Institute of Basic Medical Sciences, Chinese Academy of Medical Sciences, Beijing, China. Cells were cultured in high-glucose DMEM supplemented with 10% fetal bovine serum (FBS; Yeasen Biotechnology, Shanghai, China), 1% penicillin-streptomycin (Solarbio Science & Technology, Beijing, China), and 2 mM L-glutamine (Solarbio Science & Technology, Beijing, China) at 37 °C in a humidified atmosphere with 5% CO_2_. Normoxic culture was performed in a CO_2_ incubator (CLM-170B-8-TC; Esco (Shanghai) Enterprise Development Co., Ltd., Shanghai, China), whereas hypoxic culture was performed in a hypoxia chamber (Shenzhen Kuyuan Biotechnology Co., Ltd., Shenzhen, China).

### 4.3. In Vitro Photodynamic Therapy Protocol

For in vitro PDT, cells were incubated with 5-ALA (100 or 250 μM) or PpIX (1 μM) in serum-free DMEM for 4~6 h under hypoxic conditions (5% O_2_) to allow for photosensitizer accumulation. Prior to irradiation, the medium was replaced with PBS. A 635 ± 5 nm pumped laser source (LPS-635/660-4/4-40022-C; Lumen Photonics, Beijing, China) was used for PDT treatment. Light was delivered through a diffusing optical fiber (Laser Medical Technology, Shenzhen, China), and the light output was measured using a power meter (PM100D; Thorlabs, Newton, NJ, USA). The irradiance was then calculated based on the illuminated area. For all light exposure, irradiance was fixed at 30 mW/cm^2^. To optimize PDT conditions, cells were treated with varying concentrations of 5-ALA (0~1000 μM) and light exposure times (0, 150, or 300 s, corresponding to fluences of 0, 4.5, and 9.0 J/cm^2^). Based on the optimization results, 100 μM 5-ALA and 150 s irradiation (total dose 4.5 J/cm^2^) were selected for subsequent experiments.

### 4.4. Combined Treatment Regimens

Two distinct treatment sequences were employed to investigate the interaction between CTO and PDT.

#### 4.4.1. CTO Pre-Incubation Before PDT

To evaluate the effects of CTO exposure before PDT, cells were treated using either a “CTO→PDT→CTO” or a “CTO→PDT” regimen. For the “CTO→PDT→CTO” regimen, cells were co-incubated with CTO (10 μM) and 5-ALA (100 or 250 μM) or PpIX (1 μM) for 4~6 h before irradiation. Cells were then washed with PBS and subjected to PDT. Immediately after irradiation, the medium was replaced with complete culture medium containing 10 μM CTO and cells were further incubated for the remaining time required to achieve a total CTO exposure of 24 h. For the “CTO→PDT” regimen, cells were treated with CTO (10 μM) for 18 h and then co-incubated with CTO (10 μM) and 5-ALA for an additional 6 h. Before irradiation, the cells were washed with PBS and subjected to PDT. Immediately after irradiation, the medium was replaced with complete culture medium and cells were further incubated.

#### 4.4.2. Sequential “PDT→CTO” Treatment

Cells were first incubated with 5-ALA or PpIX for 4 h followed by PDT irradiation. Prior to irradiation, the medium was replaced with PBS. Immediately after irradiation, the medium was replaced with complete medium containing 10 μM CTO and incubated for an additional 24 h. A control group without irradiation was included to assess the non-photodynamic effect of the combined drugs.

The expected combined effect under Bliss independence was calculated as *E_Bliss_* = *E_A_* _+_ *E_B_* − *E_A_E_B_* where *E_A_* and *E_B_* represent the fractional inhibitory effects of PDT and CTO, respectively. The exploratory Bliss score was defined as the observed combination effect minus the expected effect. A positive value indicates a greater-than-additive effect at the tested condition.

### 4.5. Cell Viability Assay (SRB Assay)

Cell viability was quantified using the Sulforhodamine B (SRB) assay. The SRB assay reagents were obtained as a Cell Proliferation and Cytotoxicity Assay Kit (Yeasen Biotechnology, Shanghai, China). U87, U251 or GL261 cells were seeded in 96-well plates at a density of 1 × 10^4^ cells/well and allowed to adhere overnight. Following the specified treatments, cells from protocol 1 and 2 were assessed immediately after drug incubation, while cells from protocol 3 were assessed 18 h after PDT treatment. Cells were then fixed with cold trichloroacetic acid at 4 °C for 1 h. After washing with distilled water and air-drying, cells were stained with 0.4% (*w*/*v*) SRB solution in 1% acetic acid for 15 min at room temperature. Excess dye was removed by washing with 1% acetic acid. The protein-bound dye was subsequently dissolved in 10 mM unbuffered Tris-based solution, and the absorbance was measured at 515 nm using a microplate reader (Synergy H1; Bio Tek, Winooski, VT, USA). Cell viability was expressed as a percentage relative to the control group.

### 4.6. Cell Proliferation Assay (EdU Labeling)

The antiproliferative effects of the treatments were evaluated using a BeyoClick EdU-488 cell proliferation kit (Beyotime Biotechnology, Shanghai, China). U87 cells were seeded in 6 cm dishes at a density of 1 × 10^6^ cells/dish. Following the designated treatments, the culture medium was replaced with a medium containing 10 μM EdU and incubated for 2 h before collection. Cells were collected and fixed with 4% paraformaldehyde (Beyotime Biotechnology, Shanghai, China) for 15 min at room temperature, followed by permeabilization with a specialized buffer for 15 min. After washing with PBS, cells were incubated with 1 mL of freshly prepared Click reaction cocktail (containing CuSO_4_, Azide 488, and Click Additive Solution) for 30 min at room temperature in the dark. Samples were resuspended in 600 μL PBS and analyzed using a flow cytometer (Cytoflex; Beckman Coulter, Brea, CA, USA) at a low flow rate. The Azide 488 signal was detected using an excitation wavelength of 495 nm and an emission wavelength of 519 nm. Flow-cytometric data were analyzed using FlowJo software (version 10.8.1; BD Biosciences, San Jose, CA, USA).

### 4.7. Cell Death Analysis

Cell death was quantified by flow cytometry following Annexin V-FITC/7-AAD double-staining using a detection kit (Yeasen Biotechnology, Shanghai, China). U87 cells (1 × 10^6^ cells/dish) were harvested after hypoxia and drug treatment using 0.25% trypsin (EDTA-free; Coolaber Science & Technology, Beijing, China). The collected cells were washed with pre-cooled PBS and resuspended in 1× Binding Buffer at a concentration of 1 × 10^6^ to 5 × 10^6^ cells/mL. For each sample (100 μL), 5 μL of Annexin V-FITC was added and incubated for 5 min in the dark at room temperature, followed by the addition of 10 μL of 7-AAD and 400 μL of 1× Binding Buffer. Stained cells were filtered through a 40 μm mesh and analyzed within 1 h using flow cytometry.

### 4.8. Intracellular Total ROS Measurement

Intracellular total ROS levels were detected using the DCFH-DA fluorescent probe from a Reactive Oxygen Species Assay Kit (Yeasen Biotechnology, Shanghai, China). Treated U87 cells were harvested using EDTA-free trypsin. For the positive control group, cells were incubated with 100 mM Rosup for 30 min at 37 °C before collection. Harvested cells were incubated with 10 μM DCFH-DA in serum-free medium at a density of 1 × 10^6^ to 2 × 10^7^ cells/mL. The incubation was performed at 37 °C for 30 min in the dark, with gentle inversion every 3~5 min to ensure uniform contact. After incubation, cells were washed 1~2 times with serum-free medium to eliminate extracellular probes and finally resuspended in 600 μL PBS. The fluorescence intensity was measured using the flow cytometer described in Section 4.6 with excitation and emission wavelengths of 480 nm and 525 nm, respectively.

### 4.9. Confocal Imaging for PpIX Accumulation

To visualize the intracellular accumulation and localization of PpIX, confocal laser scanning microscopy was performed. U87 cells were seeded in confocal dishes and incubated with 100 μM 5-ALA under normoxic (21% O_2_) or hypoxic (5% O_2_) conditions for 2, 4, 6, or 8 h to evaluate the impact of oxygen levels and incubation time on PpIX production. For the co-incubation study, cells were treated simultaneously with 5-ALA and 10 μM CTO under hypoxia to assess whether CTO interfered with the metabolic kinetics of PpIX. After incubation, cells were washed three times with PBS to remove extracellular 5-ALA and fixed with 4% paraformaldehyde for 15 min. Nuclei were counterstained with DAPI (Beyotime Biotechnology, Shanghai, China) for 6 min. Images were captured using a Leica Stellar confocal laser scanning microscope (Leica Microsystems, Wetzlar, Germany). The mean fluorescence intensity of PpIX was quantified using ImageJ software (version 1.51; National Institutes of Health, Bethesda, MD, USA) and expressed as mean fluorescence intensity.

### 4.10. Mitochondrial Membrane Potential Assay

Mitochondrial membrane potential was assessed using rhodamine 123 fluorescent dye (Beyotime Biotechnology, Shanghai, China). Following the indicated treatments, U87 cells were harvested using EDTA-free trypsin. For the positive control group, cells were incubated with 10 μM CCCP (Beyotime Biotechnology, Shanghai, China) for 20 min at 37 °C before collection. The harvested cells were resuspended in staining buffer at a density of 1 × 10^6^ cells/mL and incubated with 0.5 μM rhodamine 123. The incubation was performed at 37 °C for 20 min in the dark, with gentle shaking to ensure uniform contact. After incubation, cells were washed twice with PBS to eliminate extracellular probes and finally resuspended in 600 μL PBS. The fluorescence intensity was measured via flow cytometry with excitation and emission wavelengths of 507 nm and 529 nm, respectively.

### 4.11. RNA Extraction and Quantitative Real-Time PCR (RT-qPCR)

The mRNA expression levels of genes involved in glycolysis metabolism were quantified via RT-qPCR. Total RNA was extracted from treated U87 cells using the AG RNAex Pro RNA Extraction Reagent (Accurate Biology, Changsha, Hunan, China) according to a TRIzol-based protocol. Cells were lysed in the RNA extraction reagent, and phase separation was achieved by adding chloroform (Sinopharm Chemical Reagent Co., Ltd., Shanghai, China), followed by RNA precipitation with isopropanol (Macklin Biochemical Co., Ltd., Shanghai, China) and washing with 75% ethanol (Sinopharm Chemical Reagent Co., Ltd., Shanghai, China). The RNA concentration and purity were determined using a spectrophotometer (NanoDrop One; ThermoFisher Scientific, Waltham, MA, USA). Complementary DNA (cDNA) was synthesized from 625 ng of total RNA using the Evo M-MLV Reverse Transcription Kit according to the manufacturer’s instructions (Accurate Biology, Changsha, Hunan, China). The reaction conditions were 37 °C for 15 min followed by 85 °C for 10 s. Real-time PCR was performed on a CFX Connect Real-Time PCR Detection System (Bio-Rad Laboratories, Hercules, CA, USA) using a SYBR Green Pro Taq HS qPCR kit (Accurate Biology, Changsha, Hunan, China). The reaction mixture consisted of cDNA, specific forward and reverse primers (synthesized by Tsingke Biotechnology, Beijing, China), and the qPCR master mix. The expression of glycolytic genes (*GLUT1*, *HK2*, *LDHA*) was analyzed, and the sequences of the primers used are listed in Table 1. β-actin was used as the internal control for normalization. Relative gene expression levels were calculated using the 2^−ΔΔCt^ method.

### 4.12. In Vivo Antitumor Activity

All animal procedures were conducted in accordance with the institutional guidelines for animal care. The animal experiments were approved by the Institutional Animal Care and Use Committee (IACUC) of the Institute of Basic Medical Sciences, Chinese Academy of Medical Sciences on 25 March 2024 (Approval Number: ACUC-A02-2024-015). Male BALB/c-Nude mice (SPF grade, 18~22 g) were obtained from the Animal Center of the Institute of Basic Medical Sciences, Chinese Academy of Medical Sciences, Beijing, China. To establish the subcutaneous xenograft model, 5 × 10^6^ U87 cells resuspended in 100 μL of PBS were injected into the right armpit of each mouse. When tumor volumes reached approximately 100 mm^3^, mice were randomly assigned to four groups: control, CTO, 5-ALA PDT, and 5-ALA PDT + CTO.

On day 15, the animals underwent PDT treatment. Mice were administered 5-ALA (100 mg/kg) via intraperitoneal (i.p.) injection. After 6 h of dark incubation, the tumors were exposed to external irradiation with a 635 ± 5 nm laser using the laser system described in Section 4.3 at an irradiance of 500 mW/cm^2^ for 5 min (300 s). During irradiation, the area outside the tumors was shielded. Subsequently, from the 16th day to the 22nd day, mice received CTO (90 mg/kg, prepared in PEG300) via oral gavage (i.g.) once daily. PEG300 was purchased from Solarbio Science & Technology, Beijing, China. Body weights were recorded daily, and tumor dimensions were measured using a vernier caliper. Tumor volume (V) was calculated using the formula V = (Length × Width^2^)/2. All mice were sacrificed via cervical dislocation on day 23, and tumor tissues were excised, weighed, and photographed before fixation in 4% paraformaldehyde.

### 4.13. Immunohistochemical (IHC) Analysis

To evaluate tumor cell proliferation, IHC staining was performed on paraffin-embedded sections. Excised tumor tissues were fixed in 4% paraformaldehyde, dehydrated, and embedded in paraffin. Sections were then deparaffinized in xylene (Sinopharm Chemical Reagent Co., Ltd., Shanghai, China) and rehydrated through a graded series of ethanol. Antigen retrieval was performed, followed by blocking of endogenous peroxidase activity using a 3% H_2_O_2_ solution (Sinopharm Chemical Reagent Co., Ltd., Shanghai, China) for 25 min. Non-specific binding was blocked with 3% BSA (Solarbio Science & Technology, Beijing, China) for 30 min at room temperature. Sections were incubated overnight at 4 °C with primary antibodies against Ki67 (Proteintech, Wuhan, China). After washing with PBS, sections were incubated with HRP-labeled secondary antibodies (Proteintech Group, Wuhan, Hubei, China) for 50 min. Signals were developed using a DAB chromogen kit (Servicebio, Wuhan, Hubei, China), resulting in a brown-yellow precipitate in positive areas. The sections were counterstained with hematoxylin (Servicebio, Wuhan, Hubei, China), dehydrated, and mounted for microscopic observation using a digital slide scanner (LG-FS80; Servicebio, Wuhan, Hubei, China).

### 4.14. Statistical Analysis

All experimental data are presented as the mean ± standard deviation (SD). Statistical analyses were performed using GraphPad Prism software (version 11.0.0; GraphPad Software, Boston, MA, USA). For comparisons among multiple groups, one-way analysis of variance (ANOVA) was first performed. When a significant overall difference was detected, Tukey’s post hoc test was used for multiple comparisons. The significance indicators shown in the figures are derived from these multiple comparison tests. A *p*-value of *p* < 0.05 was considered statistically significant. Schematic diagrams were created using BioGDP (https://biogdp.com/).

## 5. Conclusions

In summary, this study provides the first evidence of a sequence-dependent interaction between 5-ALA-mediated PDT and the mitochondrial inhibitor CTO in the treatment of GBM. CTO pre-incubation before PDT produced unexpected antagonistic effects, accompanied by reduced PDT-induced ROS accumulation. In contrast, a sequential “PDT→CTO” regimen yielded significantly enhanced anti-tumor efficacy both in vitro and in vivo. Mechanistically, this benefit may be associated with enhanced mitochondrial depolarization and reduced expression of glycolysis-related genes. These results highlight the importance of temporal optimization in PDT-based combination therapy and support post-PDT CTO administration as a promising strategy for targeting residual GBM cells, warranting further validation in more clinically relevant models.

## Figures and Tables

**Table 1:** Primer sequences for the qPCR experiment.

Genes	Forward Primers (5′-3′)	Reverse Primers (5′-3′)
*GLUT1*	ATGATGCGGGAGAAGAAGG	AAGACAGCGTTGATGCCAGAC
*HK2*	TTGACCAGGAGATTGACATGGG	CAACCGCATCAGGACCTCA
*LDHA*	ATGGCAACTCTAAAGGATCAGC	CCAACCCCAACAACTGTAATCT
β-actin	CATGTACGTTGCTATCCAGGC	CTCCTTAATGTCACGCACGAT

## Data Availability

The original contributions presented in this study are included in the article/Supplementary Materials. Further inquiries can be directed to the corresponding authors.

## Supplementary Materials

The following supporting information can be downloaded at: https://www.mdpi.com/article/10.3390/ijms27136091/s1.

## Abbreviations

The following abbreviations are used in this manuscript:

5-ALA	5-aminolevulinic acid
BBB	Blood–brain barrier
CAI	Carboxyamidotriazole
CTLs	Cytotoxic T lymphocytes
CTO	Carboxyamidotriazole orotate
DAMPs	Damage-associated molecular patterns
FGS	Fluorescence-guided surgery
GBM	Glioblastoma
GLUT1	Glucose transporter 1
GSH	Glutathione
HK2	Hexokinase 2
ICD	Immunogenic cell death
LDHA	Lactate dehydrogenase A
MMP	Mitochondrial membrane potential
OXPHOS	Oxidative phosphorylation
PDT	Photodynamic therapy
PpIX	Protoporphyrin IX
PS	Photosensitizer
ROS	Reactive oxygen species
SD	Standard deviation
SRB	Sulforhodamine B
TAAs	Tumor-associated antigens
TAMs	Tumor-associated macrophages
TGI	Tumor growth inhibition
TME	Tumor microenvironment
TMZ	Temozolomide
