# Supplementary material for: A Sequence-Dependent Combination of Photodynamic Therapy and Carboxyamidotriazole Orotate for Enhanced Treatment of Glioblastoma
Source: Int J Mol Sci. 2026 Jul 7;27(13):6091. doi: 10.3390/ijms27136091 (PMC13361541; doi:10.3390/ijms27136091)
Supplement: Supplementary file 1 [file ijms-27-06091-s001.zip › ijms-4325313-supplementary.pdf]

Supplementary Materials

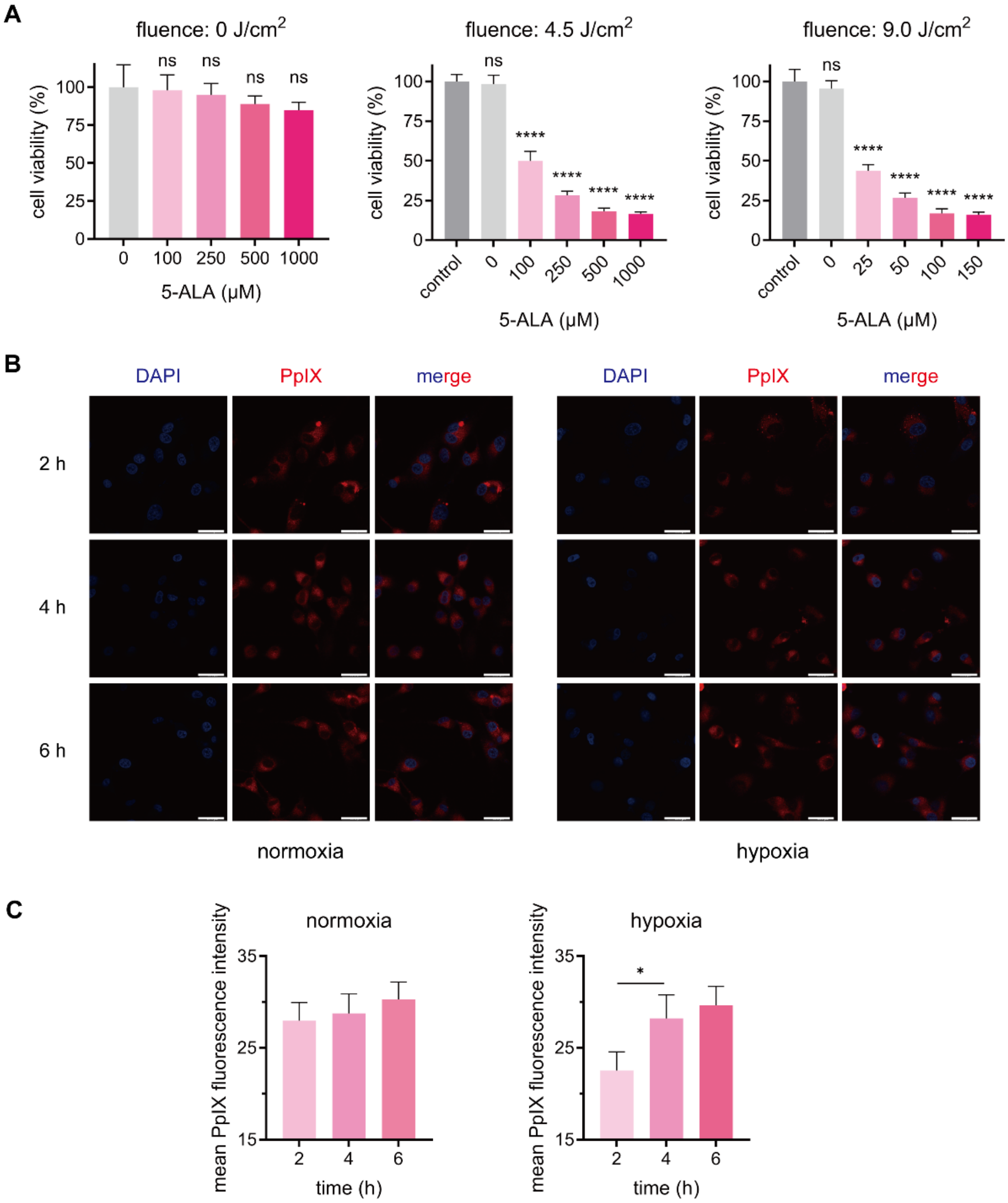

Figure S1. Optimization of PDT experimental conditions in U87 cells. (**A**) U87 cell viability assessed by SRB assay under various 5-ALA concentrations and light irradiation durations. Control groups did not receive 5-ALA or light irradiation. Cell viability was normalized to the untreated control group. (**B**) Representative confocal fluorescence images showing the intracellular accumulation of PpIX (red) in U87 cells under normoxic (21% O_2) and hypoxic (5% O_2) conditions at 2, 4, and 6 h post-incubation with 100 μM 5-ALA. Nuclei were counterstained with DAPI (blue). Scale bar: 50 μm. (**C**) Quantitative analysis of PpIX fluorescence intensity in U87 cells under the designated conditions at 2, 4 and 6 h post-incubation with 100 μM 5-ALA. Fluorescence intensity was quantified using ImageJ (version 1.51) and expressed as mean fluorescence intensity (MFI). Data are presented as mean ± SD ($n = 4$). * $p < 0.05$, **** $p < 0.0001$, ns: non-significant, compared with control (A) or between groups

connected by lines (C) (one-way ANOVA followed by Tukey's post hoc test).

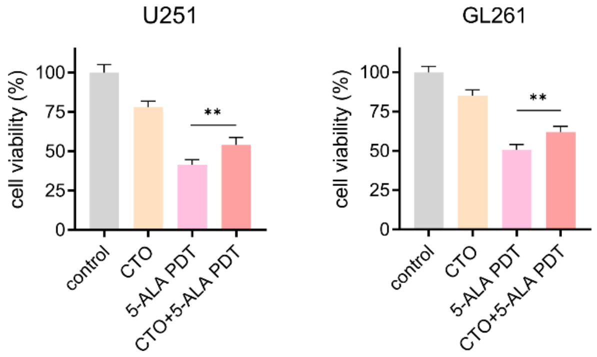

Figure S2. Cell viability of U251 and GL261 cells treated with CTO and subsequent 5-ALA PDT. Cell viability was measured by SRB assay and normalized to the untreated control group. Data are presented as mean ± SD ($n = 4$). ** $p < 0.01$ (one-way ANOVA followed by Tukey's post hoc test).

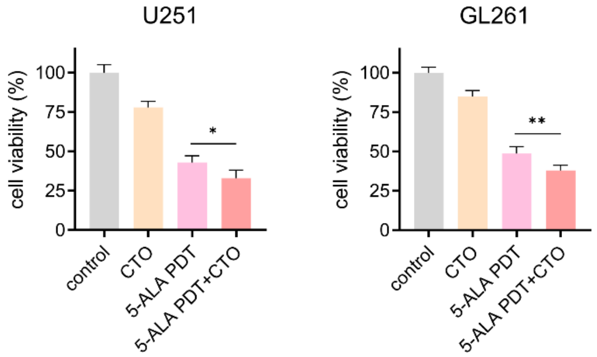

Figure S3. Cell viability of U251 and GL261 cells treated with sequential 5-ALA PDT and CTO regimen. Cell viability was measured by SRB assay and normalized to the untreated control group. Data are presented as mean ± SD ($n = 4$). * $p < 0.05$, ** $p < 0.01$ (one-way ANOVA followed by Tukey's post hoc test).

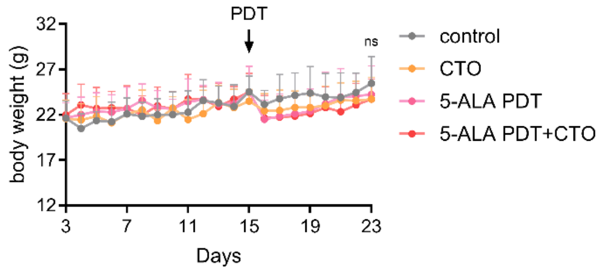

Figure S4. Average body weight of the animals during the *in vivo* anti-tumor efficacy experiment. Data are presented as mean ± SD ($n = 7$). ns: non-significant, based on body weight comparison at the endpoint (one-way ANOVA).
